# Evaluating the Efficacy of Primary Treatment for Graves' Disease Complicated by Thyrotoxic Periodic Paralysis

**DOI:** 10.1155/2014/949068

**Published:** 2014-08-03

**Authors:** Rita Yuk-Kwan Chang, Brian Hung-Hin Lang, Ai Chen Chan, Kai Pun Wong

**Affiliations:** ^1^Department of Surgery, The University of Hong Kong, Hong Kong; ^2^Division of Endocrine Surgery, Department of Surgery, Queen Mary Hospital, 102 Pokfulam Road, Hong Kong

## Abstract

*Objective*. Thyrotoxic periodic paralysis (TPP) is a potentially life-threatening complication of Graves' disease (GD). The present study compared the long-term efficacy of antithyroid drugs (ATD), radioactive iodine (RAI), and surgery in GD/TPP. *Methods*. Sixteen patients with GD/TPP were followed over a 14-year period. ATD was generally prescribed upfront for 12–18 months before RAI or surgery was considered. Outcomes such as thyrotoxic or TPP relapses were compared between the three modalities. *Results*. Eight (50.0%) patients had ATD alone, 4 (25.0%) had RAI, and 4 (25.0%) had surgery as primary treatment. Despite being able to withdraw ATD in all 8 patients for 37.5 (22–247) months, all subsequently developed thyrotoxic relapses and 4 (50.0%) had ≥1 TPP relapses. Of the four patients who had RAI, two (50%) developed thyrotoxic relapse after 12 and 29 months, respectively, and two (50.0%) became hypothyroid. The median required RAI dose to render hypothyroidism was 550 (350–700) MBq. Of the 4 patients who underwent surgery, none developed relapses but all became hypothyroid. *Conclusion*. To minimize future relapses, more definitive primary treatment such as RAI or surgery is preferred over ATD alone. If RAI is chosen over surgery, a higher dose (>550 MBq) is recommended.

## 1. Introduction

Thyrotoxic periodic paralysis (TPP) is a rare but potentially life-threatening complication of thyrotoxicosis characterized by muscle paralysis and serum hypokalemia due to massive shifting of potassium into the intracellular space [1, 2]. The overall incidence in Chinese and Japanese thyrotoxic patients has been reported to be around 2% while in the West the incidence is believed to be much lower (0.1-0.2%) [3–5]. Although any form of hyperthyroidism could potentially lead to TPP, Graves' disease (GD) is the most common cause of TPP (GD/TPP) [6]. However, unlike GD which affects mostly females, TPP predominantly occurs in males with the male to female ratio ranging from 17 : 1 to 70 : 1 [6]. During an acute attack of TPP with marked hypokalemia, cautious potassium supplementation is of paramount importance as it prevents major cardiopulmonary complications [6] but, at the same time, one must bear in mind that overadministration of potassium could lead to rebound hyperkalemia and fatal dysrhythmia during recovery from paralysis [7]. Nonselective ß-adrenergic blockade has also been proposed as an alternative to manage this acute phase and raise the serum potassium [6, 8]. Once this acute phase has been adequately dealt with, the subsequent management of GD/TPP is mostly focused on adequate control of hyperthyroidism because TPP usually does not recur once euthyroidism has been attained [6].

Despite the availability of the recent guidelines [9], the primary treatment of overt hyperthyroidism due to GD remains controversial and varies significantly in different parts of the world [6]. In our locality, the usual approach for GD with/without TPP is to use antithyroid drug (ATD) for approximately 12–18 months and then ATD is tapered or discontinued once euthyroidism has been maintained [10]. More definitive treatment modalities such as ^131^I (radioactive iodine or RAI) and surgery (in the form of total thyroidectomy) are usually reserved for relapsed thyrotoxicosis after a trial of ATD [10–12]. However, it remains unclear whether this approach or a more definitive upfront primary treatment strategy is more appropriate for GD/TPP. To our knowledge, few studies have specifically evaluated and compared the efficacy and treatment outcomes between the three primary treatment modalities (namely, ATD, RAI, and surgery) in GD/TPP. Although a large series of GD/TPP patients was reported recently, it did not specifically address this issue [13]. Thus this study aimed to evaluate and compare the efficacy and treatment outcomes of ATD, RAI, and surgery as primary treatment for GD/TPP by following the natural course of patients.

## 2. Patients and Methods

Data for this study were retrieved from the established Clinical Data Analysis and Reporting System or CDARS at our institution. CDARS is an electronic system that prospectively captures the diagnoses of all emergency admissions at our institution from the year 2000 till now. Using this database, 21 patients were retrospectively identified as having GD/TPP. For the analysis, 5 (23.8%) patients were excluded due to incomplete follow-up data. Therefore, 16 patients were analyzed. On emergency admission, all 16 patients were confirmed to be biochemically thyrotoxic at the time of paralysis, together with typical features of TPP which included history of clinically flaccid weakness or paralysis, serum potassium level <3.5 mmol/L (normal: 3.6–5.0 mmol/L), and thyrotoxicosis confirmed by serum free T4 (FT4) > 23 pmol/L (normal: 12–23 pmol/L) on presentation. In addition to these criteria, all patients with GD/TPP had to have a diffuse goiter with either clinical ophthalmopathy and/or positive antimicrosomal and/or antithyroglobulin antibodies. Anti-TSH receptor antibody (TRAb) and ultrasound (USG) were not routinely performed. Furthermore, other causes of hypokalemic periodic paralysis such as sporadic/familial hypokalemic periodic paralysis, primary aldosteronism, and renal or intestinal loss of potassium were excluded. Clinical and biochemical data during the acute episode of GD/TPP attack prior to initiation of potassium supplement including blood pressure, heart rate, serum potassium, thyroid-stimulating hormone (TSH) and FT4 levels, primary treatment modality, and subsequent treatment outcomes such as thyrotoxic relapse, latest thyroid function status, and surgical-related complications were recorded.

### 2.1. Treatment Principles of GD/TPP

Details of primary treatment of GD were described previously [10]. In general, as a primary treatment of GD with/without TPP, patients were prescribed a course of ATDs (usually in the form of carbimazole (CMZ) or propylthiouracil (PTU)) lasting for a period of 12–18 months and then thereafter tapered or stopped when thyroid function was normal. A great proportion of GD would eventually go into prolonged remission after ATD withdrawal [11]. If there was evidence of thyrotoxic relapse after a full course of ATDs, definitive treatment such as RAI or total thyroidectomy was offered, depending on goiter size and patient's preference. Giving another course of ATDs after a thyrotoxic relapse was an alternative if the patient was not keen on or was contraindicated to RAI or surgery. Those who underwent RAI or surgery were rendered euthyroid by ATDs +/− Lugol's solution before procedure. After primary treatment, all patients were regularly followed up for monitoring of thyroid function. The clinical course of these patients was followed to the time of analysis. Follow-up period was expressed in months and was calculated from the diagnosis of GD (with or without TPP) to the latest follow-up.

All statistical analyses were performed using SPSS version 18.0 (SPSS, Inc., Chicago, IL, USA). Chi-square or Fisher's Exact test was used to compare categorical variables.

## 3. Results

Table 1 shows the demographic features of 16 TPP patients. The median (range) age on presentation was 28 (17–45) years old. There were 15 males and 1 female. The median (range) body weight was 69.9 (45–105) kg. Among the cohort, 4 (25.0%) had family history of GD. Thirteen (81.2%) had no previous history of thyroid disease and TPP was the first presenting sign of thyrotoxicosis. Two (12.5%) had associated Graves' ophthalmopathy. These features were not significantly different when comparing patients who received ATD, RAI, or surgery as the primary treatment for GD/TPP. Among our 16 patients, 8 (50.0%) had ATD, 4 (25.0%) had RAI, and 4 (25.0%) had surgery as the primary treatment for GD/TPP. There were 10 (62.5%) thyrotoxic recurrences. When comparing different treatment modalities, all 8 patients who received ATD had thyrotoxicosis recurrence (100%), while 2 out of 4 (50%) in RAI group and 0% in the surgery group had thyrotoxic relapse (*P* = 0.003). Regarding the recurrence of TPP, the overall recurrence rate was 31% (5 patients) after primary treatment.

Table 2 summarizes the clinical and biochemical profiles of the 16 patients on the presentation of GD/TPP. During the acute attack of TPP, all patients presented bilateral lower limb flaccid weaknesses, hypokalemia with serum potassium of 2.5 mmol/L (1.2–3.4) mmol/L, and thyrotoxicosis (TSH 0.03 (range: 0.01–0.18) mU/L and fT4 60.4 (range: 24.1–140) ug/dL) (Table 2). All of them had normal body temperature and pulse oximeter reading. Electrocardiographic changes (T inversion in lead II to V) were found in 1 patient with preexisting heart disease. Three (18.8%) patients had paralysis upon waking up in early morning. Regarding precipitating factors, TPP occurred after taking a heavy meal (*n* = 4) or alcohol (*n* = 3). Other factors included acute upper respiratory infection (*n* = 2) and strenuous exercise (*n* = 1), while 7 patients had no specific triggers identified. None of these patients had symptoms and signs involving respiratory system and bulbar and ocular muscles during GD/TPP. Symptoms of TPP subsided spontaneously in 2 of our patients, while the rest required potassium supplementation to reverse muscle weakness. None of them were prescribed ß-blockers at acute attack.

### 3.1. Details of Treatment Outcomes

Table 3 shows the detailed clinical course of these 16 patients. The median (range) follow-up period was 91.9 (11.2–293.2) months. In the ATD group, all 8 patients completed their course with a median duration of 19.5 (12–24) months. Although these 8 patients were able to have ATD withdrawn for a median duration of 37.5 (22–247) months, they all developed thyrotoxic relapses subsequently. Among these 8 patients, 4 (50.0%) patients suffered at least one further attack of TPP during their thyrotoxic relapses (patients 2, 3, 6, and 7). Three patients with thyrotoxic relapses eventually underwent total thyroidectomy (patients 1, 4, and 8) while the other 3 required 2 courses of RAI to render remission (patients 5, 6, and 7). In this group, the median accumulative RAI dose to render hypothyroidism was 550 (500–700) MBq. All 3 patients required daily thyroxine replacement. In the ATD group, 2 (25.0%) patients (patients 2 and 3) remained hyperthyroid and were on ATD on latest follow-up because they remained reluctant to undergo RAI or surgery.

Of the four patients who received RAI as the primary treatment (patients 9–12), two (50%) developed thyrotoxic relapse in 12 and 29 months, respectively. The median first RAI dose given was 325 (300–350) MBq while the median accumulative RAI dose required to render hypothyroidism was 550 (350–700) MBq. Of the two relapses after RAI, one (patient 9) had a further 12-month course of ATD and subsequently remained euthyroid at the latest follow-up. The other patient with thyrotoxic relapse received a further 300Mbq RAI and was hypothyroid requiring thyroxine replacement at the latest follow-up (patient 10). In summary, among the four patients who received RAI as primary treatment, two (50.0%) developed thyrotoxic relapse requiring further treatment and 2 (50.0%) developed hypothyroidism requiring thyroxine replacement.

Table 4 shows the details of 7 patients with GD/TPP who eventually underwent surgery. Of these 7 patients, 3 patients (patients 1, 4, and 8) were initially treated with a trial of ATD before developing thyrotoxic relapse. Four (25.0%) patients underwent total thyroidectomy as primary treatment (patients 13–15). All patients recovered well after total thyroidectomy without the need for calcium +/− vitamin D supplement. None of the patients had vocal cord paresis as documented by postoperative laryngoscopy. All 7 patients were placed on thyroxine replacement therapy with stable thyroid function. None of them developed relapsed thyrotoxicosis or TPP at the latest follow-up.

## 4. Discussion

TPP is a rare but potentially lethal complication of hyperthyroidism characterized by muscle paralysis and hypokalemia [6]. Our series demonstrated the classical features of TPP with the disease predominantly affecting young males of Asian descent. Over 80% of our patients presented with TPP as the first presenting symptom for thyrotoxicosis. This highlights the importance of thinking about diagnosis of TPP and assessing thyroid function in a young Asian male with hypokalemic periodic flaccid paralysis [3–6].

One of the interesting findings from this observational study was that despite the fact that the 8 patients with GD/TPP were treated adequately with ATD for a median duration of 19.5 months, all developed thyrotoxic relapse after ATD withdrawal for a variable period of time. In the literature, it is unclear what the actual thyrotoxic relapse rate for GD/TPP treated with ATD is, but for GD in general (i.e., with or without TPP), the well-quoted figure for relapse after ATD is 50–60% [12, 14]. Although the high relapse rate in our study might be an overestimation as a result of selection biases or small sample size, it seems that GD/TPP might be less responsive to ATD alone when compared to GD in general. It is possible that the occurrence of TPP may signify a more severe entity of GD which is less likely to go into remission with ATD alone. Of course this postulation requires larger prospective studies to verify.

Another finding worth noting was that among the 8 patients who eventually relapsed after ATD, four (50%) suffered another episode of paralysis. This TPP relapse rate appeared higher than a recent study which reported 29.6% of patients had repeated TPP during withdrawal or tapering of ATD [13]. However, this discrepancy might be due to the difference in the length of follow-up between the two studies as all of those on ATD were followed up for over 5 years (see Table 3). Nevertheless, these data suggested that significant proportion of thyrotoxic relapse in GD/TPP after treatment by ATD would be complicated by TPP. Moreover, giving repeated courses of ATD after ATD treatment failure did not appear to be effective in achieving remission in GD/TPP. Two patients (patients 2 and 3) remained hyperthyroid requiring ATD after a follow-up period of 86.3 and 124.8 months, respectively, while the rest ultimately required definitive treatment. This observation therefore suggested that either RAI or surgery might be a better primary treatment modality than ATD to achieve remission and to prevent relapse for GD/TPP in the long term.

However, it is worth noting that the relapse rate after giving RAI was also high. Within the 7 patients who received RAI for the first time (i.e., including those who relapsed with ATD), 5 developed thyrotoxic relapses (patients 5, 6, 7, 9, and 10) and, of these, 3 had TPP during the relapse (patients 6, 7, and 9). One possible reason for this high relapse rate might be related to the low RAI dose used. Of the two patients receiving RAI 250 MBq, all (100.0%) had thyrotoxic relapse while for the three patients receiving RAI 350 MBq, two out of three (66.7%) had relapse. In this study, an accumulative RAI dose ≥500 MBq would render patients permanently hypothyroid. This finding appeared comparable to previous studies [15, 16]. Thus a higher dose of RAI should be considered to achieve cure in GD/TPP [17] with an understanding that this approach will most likely lead to permanent hypothyroidism requiring T4 replacement. However since RAI could potentially cause transient hyperthyroidism and perhaps early TPP relapse [18], caution should be exercised when RAI is chosen as definitive treatment. Perhaps, RAI should be avoided if a patient has or is at risk of cardiovascular disease or significant arrhythmias [18].

Since total thyroidectomy involves removal of almost all thyroid tissue, it is expected that relapse would be rare. In our experience, all 7 patients with GD/TPP who underwent total thyroidectomy had an uneventful recovery with no significant complications. Although all patients required life-long thyroxine replacement after surgery, thyroid function was easier to manage. Interestingly, rate of hypoparathyroidism appeared comparatively lower in this group of patients [19].

Despite these findings, we would like to acknowledge several shortcomings. Firstly, since this was a retrospective analysis that relied on the accuracy of a prospectively collected database, selection biases were inevitable. For example, patients with thyrotoxic or TPP relapses might have been more likely captured by the CDARS database than those without relapses because the former would have had multiple admissions and a greater chance of being captured by the database. Moreover, some important data such as TRAb titre, goiter volume by USG, and degree of ophthalmopathy were not routinely checked in our center but could be significant factors for disease relapses. Thirdly, despite a relatively long study period, there were few patients eligible for analysis and so this study might have been underpowered to detect smaller differences.

## 5. Conclusion

Patients with GD/TPP appeared to have a greater chance of thyrotoxic and paralysis relapse when managed by ATD alone. As a result, clinicians should consider more definitive treatment such as surgery or RAI as the primary treatment modality for patients with GD/TPP. If RAI is chosen over surgery, a higher dose (>550 MBq) should be used to minimize future relapses with an understanding that permanent hypothyroidism might be inevitable.

## Figures and Tables

**Table 1:** A comparison of patient demographics and treatment outcome between patients who had antithyroid drugs (ATD), radioactive iodine (RAI), and surgery as the primary treatment for Graves' disease with thyrotoxic periodic paralysis (TPP).

	Number (%)/median (range)				*P* value
	Total (*n* = 16)	ATD (*n* = 8)	RAI (*n* = 4)	Surgery (*n* = 4)	
Gender (male : female)	15 : 1	8 : 0	4 : 0	3 : 1	0.202
Age (years)	28 (17–45)	26.0 (17–33)	36 (23–42)	30.5 (26–45)	—
Smoker	10 (62.5%)	4 (50%)	2 (50%)	4 (100%)	0.202
Drinker	6 (37.5%)	2 (25%)	1 (25%)	3 (75%)	0.202
Body weight (kg)	69.9 (45.0–105)	70.9 (61–87)	66.5 (63–105)	74.5 (45–82)	—
Family history of thyrotoxicosis	4 (25.0%)	1 (12.5%)	2 (50.0%)	1 (25%)	0.368
TPP as the initial thyrotoxicosis symptom	13 (81.3%)	5 (62.5%)	4 (100%)	4 (100%)	0.158
Presence of severe Graves' ophthalmopathy∗	2 (12.5%)	1 (12.5%)	0 (0.0%)	1 (25%)	0.565
Antithyroglobulin antibodies (<100 L/titre)	1088 (63–6400)	413 (63–1600)	3276 (100–6400)	81 (63–100)	—
Antithyroid microsomal antibodies (<100 L/titre)	3325 (100–6400)	300 (100–400)	6400 (6400–6400)	3250 (100–6400)	—
Outcome after primary treatment					
Recurrence of thyrotoxicosis	10 (62.5%)	8 (100%)	2 (50%)	0 (0%)	**0.003**
Recurrence of TPP	4 (25.0%)	4 (50%)	0 (0%)	0 (0%)	0.069
Hypothyroidism requiring replacement	12 (75.0%)	6 (75.0%)	2 (50%)	4 (100%)	0.264

*Although all patients had mild eye signs, only two patients had severe eye signs and required ophthalmic treatment for their eye condition.

**Table 2:** Clinical and biochemical profiles of the 16 patients on the presentation of GD/TPP.

	Number (%)/median (range)
Systolic blood pressure (mmHg)	131 (90–154)
Diastolic blood pressure (mmHg)	77 (69–99)
Heart rate (beats/min)	100 (66–138)
Body temperature (°C)	37.0 (36.7–37.2)
TSH (normal: 0.35–4.78 mIU/L)	0.03 (0.01–0.18)
Free T4 (normal: 12–23 pmol/L)	57.5 (24.1–140)
K^+^ (normal: 3.6–5.0 mmol/L)	2.7 (1.2–3.4)
Precipitating/exacerbating factors	
Heavy meals	4 (25.0%)
Alcohol intake	3 (18.8%)
Acute upper respiratory infection	2 (12.5%)
Strenuous exercise	1 (6.3%)
No factor identified	7 (43.8%)

TSH: thyroid stimulating hormone; K^+^: serum potassium.

**Table 3:** A summary of the clinical course of the 16 patients with thyrotoxic periodic paralysis secondary to Graves' disease (TPP/GD).

Patient number	Sex/age (years)	Primary treatment modality	Interval to first thyrotoxic relapse (months)	Treatment for first thyrotoxic relapse	Interval to second thyrotoxic relapse (months)	Treatment for second thyrotoxic relapse	Follow-up period (months)	Thyroid function status on latest follow-up
1∗	M/31	ATD	22	ATD	25	Surgery	68.0	T4
2	M/33	ATD	76^#^	ATD	20	ATD	124.8	Hyperthyroid
3∗	M/17	ATD	35^#^	ATD	23^†^	ATD	86.3	Hyperthyroid
4∗	M/25	ATD	40	ATD	52	Surgery	113.7	T4
5∗	M/32	ATD	28	RAI 250 MBq	12	RAI 250 MBq	154.3	T4
6	M/23	ATD	247^#^	RAI 350 MBq	6^†^	RAI 350 MBq	293.2	T4
7	M/27	ATD	102^#^	RAI 250 MBq	5^†^	RAI 300 MBq	146.1	T4
8∗	M/24	ATD	29	Surgery	—	—	66.0	T4
9∗	M/33	RAI 300 MBq	29^#^	ATD	—	—	87.3	Euthyroid
10∗	M/23	RAI 350 MBq	12	RAI 300 MBq	—	—	49.7	T4
11∗	M/39	RAI 300 MBq	—	—	—	—	22.4	Euthyroid
12∗	M/42	RAI 350 MBq	—	—	—	—	96.5	T4
13∗	M/45	Surgery	—	—	—	—	11.2	T4
14∗	F/26	Surgery	—	—	—	—	152.2	T4
15∗	M/32	Surgery	—	—	—	—	19.3	T4
16	M/39	Surgery	—	—	—	—	125.6	T4

GD/TPP: thyrotoxic periodic paralysis secondary to Graves' disease; ATD: antithyroid drugs; RAI: ^131^I or radioactive iodine; T4: requiring daily thyroxine replacement.

Note: the primary treatment of patient 7 was switched to PTU due to allergy to CMZ.

*Where TPP was the first presenting symptom of thyrotoxicosis.

^#^Patient who had an attack of TPP during the first thyrotoxic relapse.

^†^Patient who had an attack of TPP during the second thyrotoxic relapse.

**Table 4:** Details of the 7 patients with thyrotoxic periodic paralysis who eventually underwent surgery/total thyroidectomy.

Patient number	Sex/age (years)	Surgical indication	Number of thyrotoxic relapses before surgery	Weight of excised thyroid gland (g)	Operating time (minutes)	Blood loss (mL)	Hospital stay (days)	RLN injury	Postoperative hypocalcemia
1	M/31	Patient preference	2	40	69	10	2	nil	nil
4	M/25	Patient preference	2	86	151	200	2	nil	nil
8	M/24	Patient preference	1	45	58	10	2	nil	nil
13	M/45	Large goiter	0	125	273	300	3	nil	nil
14	F/26	Patient preference	0	36	165	5	2	nil	nil
15	M/32	Patient preference	0	33	90	3	2	nil	nil
16	M/39	Large goiter	0	78	144	60	3	nil	nil

RLN: recurrent laryngeal nerve.
